# TiO_2_-Based Nanoheterostructures for Promoting Gas Sensitivity Performance: Designs, Developments, and Prospects

**DOI:** 10.3390/s17091971

**Published:** 2017-08-27

**Authors:** Yuan Wang, Tao Wu, Yun Zhou, Chuanmin Meng, Wenjun Zhu, Lixin Liu

**Affiliations:** 1 National Key Laboratory of Shock Wave and Detonation Physics, Institute of Fluid Physics, China Academy of Engineering Physics, PO Box 919-111, Mianyang 621900, Sichuan, China; tomasiwt@gmail.com (T.W.); zhouyun720@126.com (Y.Z.); mcm901570@126.com (C.M.); wjzhu@caep.ac.cn (W.Z.); 2 School of National Defense Science and Technology, Southwest University for Science and Technology, Mianyang 621900, Sichuan, China; 3 School of Materials Science and Engineering, Xiangtan University, Xiangtan 411105, Hunan, China

**Keywords:** TiO_2_, nanoheterostructures, gas sensor

## Abstract

Gas sensors based on titanium dioxide (TiO_2_) have attracted much public attention during the past decades due to their excellent potential for applications in environmental pollution remediation, transportation industries, personal safety, biology, and medicine. Numerous efforts have therefore been devoted to improving the sensing performance of TiO_2_. In those effects, the construct of nanoheterostructures is a promising tactic in gas sensing modification, which shows superior sensing performance to that of the single component-based sensors. In this review, we briefly summarize and highlight the development of TiO_2_-based heterostructure gas sensing materials with diverse models, including semiconductor/semiconductor nanoheterostructures, noble metal/semiconductor nanoheterostructures, carbon-group-materials/semiconductor nano- heterostructures, and organic/inorganic nanoheterostructures, which have been investigated for effective enhancement of gas sensing properties through the increase of sensitivity, selectivity, and stability, decrease of optimal work temperature and response/recovery time, and minimization of detectable levels.

## 1. Introduction

Since the 20th century, atmospheric pollution has been proved to be one of most urgent issues. For the sake of controlling the exhaust emissions, gas sensors for the quantitative detection of various toxic and harmful gases have been widely developed as a result of their high response, outstanding selectivity, excellent repeatability, and good stability [1,2,3]. So far a variety of gas sensors, such as metal oxide semiconductor-based gas sensors [4,5,6,7,8,9], solid electrolyte-based gas sensors [10], electrochemical gas sensors [11], carbon-based gas sensors [1,12,13,14], organic gas sensors [2,3], and so on, have been extensively investigated. Amongst these different types of gas sensors, resistance type metal oxide gas sensors offering low cost, simple manufacturing approaches, and excellent sensitivity to the great majority of gases, have attracted considerable attention during the past several years [15,16]. Since Seiyama [17] reported metal oxide-based gas sensors for the first time, a large amount of effort has been expended in exploring the sensing properties of metal oxide- based gas sensors [7,18,19].

As a representative semiconductor metal oxide, titanium dioxide (TiO_2_) has attracted much attention since Fujishima et al. observed the photocatalytic splitting of water on a TiO_2_ electrode under the irradiation of UV light in 1972 [20]. In the past decades, it has been discovered that TiO_2_ could be employed in many promising fields including photovoltaics [21], photocatalysis [22,23], sensors, etc. [24,25,26]. In particular, because of its high stability, harsh environmental tolerance, and environmentally-friendly properties, TiO_2_ has been widely investigated and is regarded as one of sensing materials for gas detection with the most potential [27,28,29,30].

**Figure 1:**
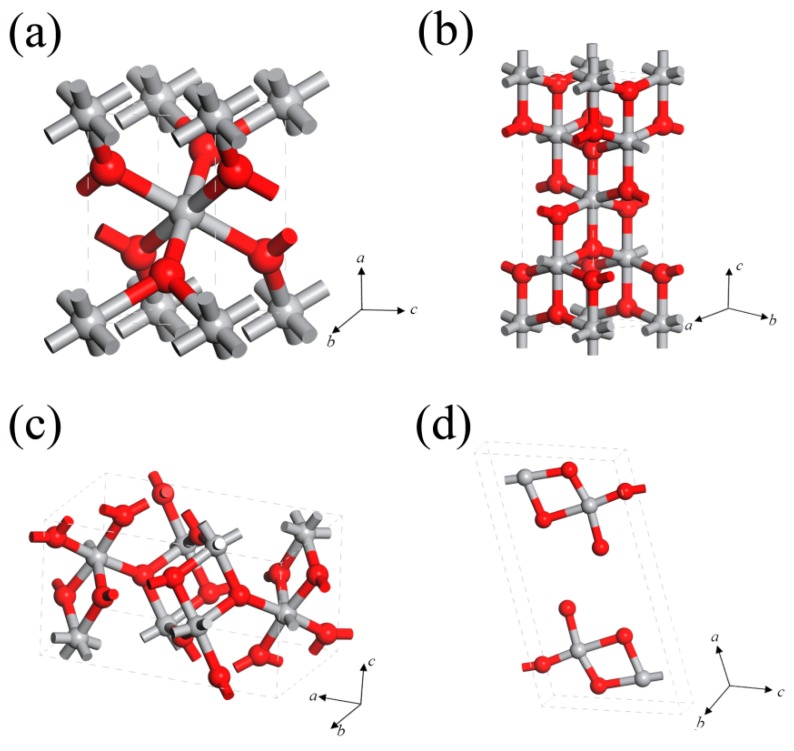
Crystal structures of TiO_2_: (**a**) Rutile; (**b**) Anatase; (**c**) Brookite; and (**d**) TiO_2_(B), red spheres represent Ti atoms, and the grey spheres represent O atoms.

Naturally, TiO_2_ mainly exists in three polymorph forms: rutile phase (tetragonal, P42/mnm) [31], anatase phase (tetragonal, I41/amd), and brookite phase (orthorhombic, pbca) (Figure 1a–c), whose bandgaps are 3.02, 3.2, and 2.96 eV, respectively [32]. Besides the abovementioned three crystal phases, there exists another phase, TiO_2_(B) (monoclinic, C2/m). As shown in Figure 1d, TiO_2_(B) possesses a layer structure, thus the density is lower and the specific capacity is larger comparing with other phases [33,34,35]. Among these diverse crystal phases, the most stable bulk phase is rutile, whereas for nanomaterials, anatase and brookite are commonly recognized to be more stable phases because of their relatively lower surface energy than rutile, although this fact is still argued in previous reports [36,37]. In the practical applications, TiO_2_ performance is commonly affected by the crystal phases, which can be obtained through controlling the experimental conditions, such as fabrication methods, pH, duration annealing, temperature, and so on. As for the sensing applications, rutile TiO_2_ and anatase TiO_2_ are the most studied polymorphs.

**Figure 2:**
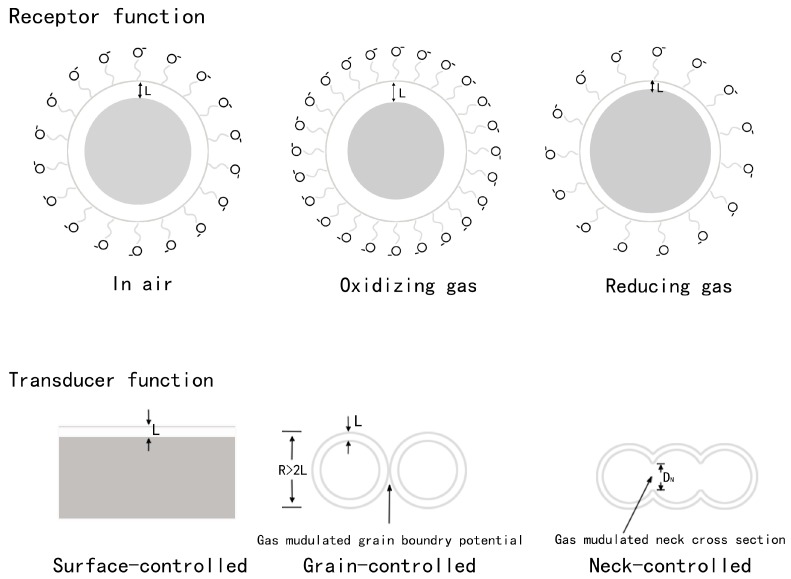
Schematic image of gas sensing at different modes, where L represents the depletion layer, R represents particle size, and D_N_ represents the diameter of the neck cross section.

TiO_2_ gas sensors are typical resistant-type sensors which can display a decrease or increase in resistance when probing a reductive gas (H_2_, H_2_S, NH_3_, CO, VOCs) or oxidative gas (NO_2_, O_2_) [29,30,38], respectively. The sensing mechanism of TiO_2_-based gas sensors can be described by the following two-step process: receptor process and transducer process, as shown in Figure 2 [39].

The receptor process occurs at the TiO_2_ surface, and it involves physisorption and chemisorption processes [40]. Firstly, oxygen molecules can be physically absorbed on the surface when TiO_2_ is exposed to an air environment at room temperature; the process is determined by Van der Waals and dipole interactions; secondly, oxygen molecules on the TiO_2_ surface will capture electrons from the conductive band (CB) of TiO_2_ to form chemisorbed oxygen species (O_2_^−^) on the surface. The reactions taking place on the surface of TiO_2_ are as follows:

(1) $$O_{2}\left(g\right)\to O_{2}\left(ads\right)$$

(2) $$O_{2}\left(ads\right)+e\to O_{2}^{-}$$

During the process, receptor capability is determined by the physisorption process and chemisorption process together, where the physisorption can be influenced by the temperature, whereas the rate of chemisorption process may be influenced by activation energy.

The transducer process includes the transportation of electrons in the TiO_2_ and the transformation of electrons into the outward resistance signal. This can be influenced by the three typical electron transfer modes which are divided as surface-controlled mode, grain-controlled mode, and neck-controlled mode, respectively, [41,42] as shown in Figure 2. As for surface-controlled mode, compact layer structures determined by the thin film thickness of the materials are universally considered as the main pattern [39], where the gases can only affect the materials surfaces other than the internal body. On the contrary, in real polycrystalline materials, the TiO_2_ grains connect to each other through grain boundaries or necks, in this way, the grain boundaries or necks will contribute significantly to the electroconductibility and gas sensing performance of the TiO_2_. It has been reported that materials with large grain size would possess large neck cross sections, so accordingly the neck resistance is less significant than the grain-boundary resistance. However, for materials with much smaller grain size, the neck resistance is higher than the grain boundary resistance because of the much smaller neck cross section, in this case the neck resistance becomes more significant [41].

In these two typical processes presented above, surface-to-volume ratio, grain size, and the electron transport ability of TiO_2_-based materials play important roles. Consequently, in recent years, substantial effort has been invested in increasing the specific surface area, decreasing the grain size, and enhancing the conductivity of TiO_2_ through nanostructured materials, element doping, heterostructural materials and so on [27,30,43,44,45,46].

Nano-scale is a key factor in studying the gas sensing properties of metal oxide semiconductor-s based sensors. In fact, it is well known that the surface structure and specific surface area can play very important roles in sensing properties [47]. Nanocrystallization is an efficient way to improve the gas sensing performance because of their much larger specific surface area and rich surface chemical properties on the nanostructure surfaces, which may potentially lead to miniaturized sensors with outstanding performance. In this regard, many kinds of TiO_2_ nanostructures with various morphologies, such as zero-dimensional (0D) nanocrystals [48], 1D nanofibers or nanowires [43,49,50], 2D nanoplates [29], and 3D hierarchical microstructures [27] have been developed.

Another important strategy to enhance the sensing performance of TiO_2_ is the formation of heterostructures. The heterojunction theory dates back to as early as the 1930s [51]. Since then, more and more applications of nanoheterojunctions have been extensively investigated owning to the superinjection of charge carriers. Since 2005, a great many nanoheterostructural materials have been widely researched and applied in many fields, including solar cells [52], Li-ion batteries [53], photoelectrochemical cells [54], photocatalysis [55], and gas sensors [5,56]. As a matter of fact, several TiO_2_-based nanoheterostructures, such as semiconductor/semiconductor, noble metal/ semiconductor, carbon-group-material/semiconductor and organic/inorganic nanoheterostructures, have attracted widespread interest in the preparation and investigation of their properties and applications. Various types of TiO_2_-based nanoheterostructures including composite nanoparticles [57,58,59], quantum dots in nanowires/nanofibers [60,61,62,63], core/shell nanowires/nanofibers/ nanospheres [64,65], etc., have been studied intensively. Compared with the pure oxide, these nanoheterostructures achieve higher sensing performance. As a well-known strategy, the modification of TiO_2_ nanostructures using noble metal nanoparticles, like platinum (Pt), palladium (Pd), silver (Ag), and gold (Au), forming noble metal/TiO_2_ heterostructured sensor materials can further improve the sensing performance, including enhancement of sensitivity and selectivity and shortening of response/recovery times [66,67]. The enhanced properties can be generally ascribed to the catalytic activity of noble metal nanoparticles, increased active surface area, reduced electrical resistance, enhanced optical absorption, facilitated chemical adsorption of oxygen molecules and/or reaction of oxygen ions with probing gases, and improved gas diffusion inside the heterostructures [68]. In addition, the Schottky barrier formed at the interface of noble metal/TiO_2_ heterojunction yielding efficient electron/hole separation is considered as another important factor in enhancing sensing performance. In general, the electronic and chemical properties of the metal/oxide interface, as well as the morphology of the noble metal nanoparticles and metal oxides matrix, play important roles in promoting the overall performance of the noble metal/TiO_2_ nanoheterostructures [57,60,69,70]. Comprehensive coverage of metal/semiconductor nanoheterostructured sensors have been available elsewhere for many decades, so accordingly, in this review, we briefly trace the application of TiO_2_-based semiconductor/semiconductor, carbon-group-material/semiconductor and organic/inorganic nanoheterostructures in the area of gas sensors, summarize their major design and preparation methods, describe some of the improvements and resulting achievements, and discuss the future challenges and perspectives.

## 2. Fabrication of TiO_2_-Based Nanoheterostructures

In the past few decades, various fabrication methods, including chemical vapor deposition (CVD), atomic layer deposition (ALD), solid phase reaction, electrochemical deposition, chemical deposition, hydrothermal/solvothermal methods, sol-gel, electrospinning, etc., have rapidly developed and been successfully applied in preparing high quality TiO_2_-based nanoheterostructures. Accordingly, in this section, most of the attention will be focused on the nanoheterostructure synthesis methods.

### 2.1. CVD and ALD Methods

The CVD route probably is one of most extensively explored approach in nanoheterostructure preparation, which can deposit various materials onto suitable substrates in order. Nano- heterostructures are generally synthesized using a two-step growth procedure. In the first step, the inner-core material is deposited on a suitable substrate through CVD or other synthetic routes. In the second step, the outer-layer shell material is subsequently grown on the core surface. This method has the capability to control the components, morphology, thickness, and length of the materials by controlling some technological parameters including temperature, pressure, carrier gases, gas-flow rates, substrates, and deposition time. For example, ZnO-TiO_2_ nanocomposites were fabricated via an innovative CVD technique [46], where TiO_2_ nanoparticles were grown on the initially deposited ZnO nanoplatelet host. The process was carried out at a relatively low temperature of 350–400 °C, this avoiding the effect of unsuitable thermal treatment and maintaining the chemical properties of the materials. Barreca et al. [71] have reported the fabrication of CuO-TiO_2_ nanocomposites through a multistep vapor deposition process, where the first step was the synthesis of porous CuO nanomaterials on an Al_2_O_3_ substrate via a CVD approach, and the final step was the controllable growth of TiO_2_ nanoparticles on the porous CuO matrices.

**Figure 3:**
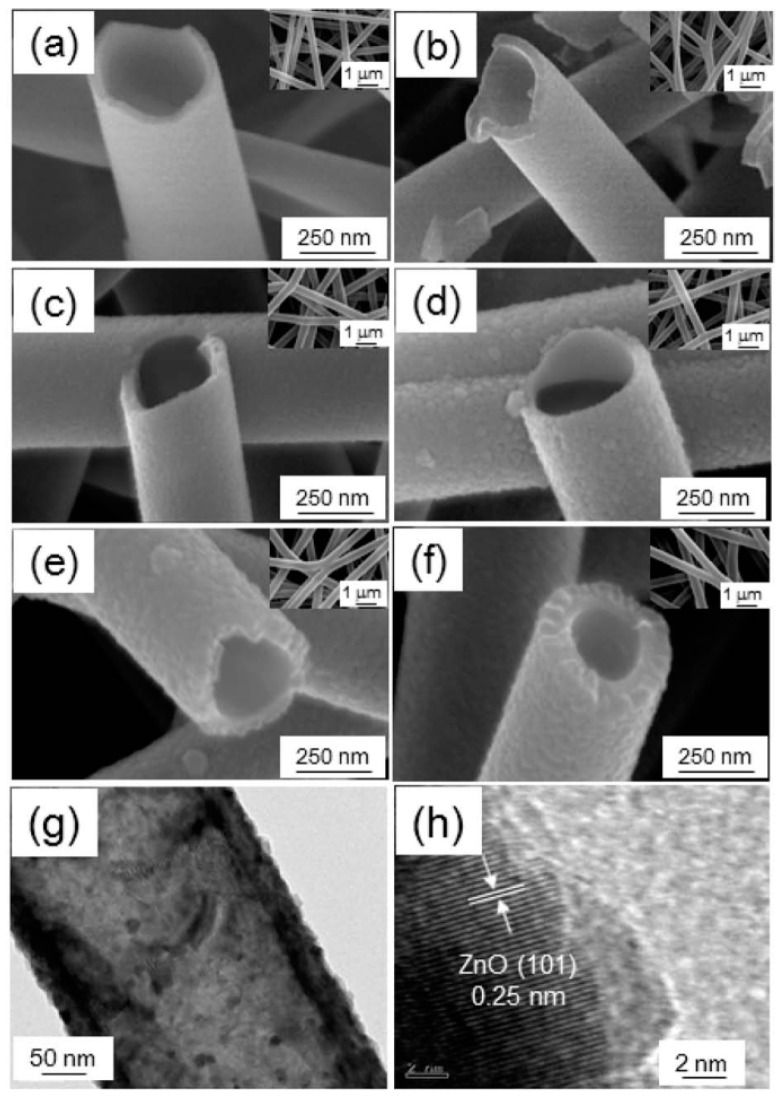
(**a**–**f**) Scanning electron microscope (SEM) images of TiO_2_ hollow fibers synthesized with 1000 ALD cycles (**a**); TiO_2_/ZnO double-layer hollow fibers synthesized with 20 ALD cycles (**b**); 50 ALD cycles (**c**); 90 ALD cycles (**d**); 220 ALD cycles (**e**); and 350 ALD cycles (**f**); (**g**) Transmission electron microscope (TEM) image of a single TiO_2_/ZnO DLHF; (**h**) High resolution transmission electron microscopy (HRTEM) image of the outer layer ZnO [72]. Copyright 2014 American Chemical Society.

Although involving a similar chemical process as the CVD route, ALD has attracted much attention in the synthesis of heterostructures because of the accurate control of film thickness at atomic scale and the conformal growth of complex nanostructures. The excellent conformability between two materials obtained by ALD makes them possible to form heterojunctions at the semiconductor interfaces. Through growth control, Katoch et al. [72] have successfully prepared TiO_2_/ZnO inner/outer double-layer hollow fibers (TiO_2_/ZnO DLHFs), as shown in Figure 3. The TiO_2_/ZnO DLHFs were synthesized using a three-step process. First, polyvinyl acetate (PVA) fibers were prepared by an electrospinning process; subsequently, TiO_2_ and ZnO were sequentially grown on the PVA fibers through the ALD method and, finally, a thermal treatment was carried out for the removal of the PVA support and the crystallization of TiO_2_ and ZnO.

### 2.2. Solid Phase Reactions

Solid phase reactions are a quite facile process to synthesize composite sensing materials. In a typical process, pure powders are mixed uniformly using a physical method to electrostatically self-assemble nanoheterostructures, which generally is a one-step procedure. For example, Zhou et al. have prepared Ag_2_O/TiO_2_ nanoheterostructures [73] through a solid phase reaction, where a certain amount of the pure Ag_2_O and the corresponding amount of TiO_2_ were uniformly mixed to get Ag_2_O/TiO_2_ nanoheterostructures. What’s more, some other nanoheterostructures, such as polyaniline-titanium (PANi-TiO_2_) nanoheterostructures [74], reduced graphene oxide/titanium dioxide (rGO/TiO_2_) layered nanofilm [75], ZnO–TiO_2_ nanocomposites [59], and TiO_2_/SnO_2_ nanocomposites [76], have also been successfully synthesized using this facile method.

### 2.3. Electrochemical Deposition

Compared with the CVD route, electrochemical deposition can obtain large-scale nanostructures at relatively low temperature. The conventional technique is very propitious to the fabrication of ordered, uniform, and highly dense nanoheterostructures for widespread applications. Recent years, some types of TiO_2_-based nanoheterostructures are prepared using a two-step electrochemical deposition process [60,77]. In the deposition process, the pre-grown nanomaterials on fluorine-doped tin oxide (FTO), indium-doped tin oxide (ITO), or other relevant substrates are treated as working electrode for the following deposition. For instance, Yang and co-workers [78] have reported the preparation of the Cu-Cu_2_O/TiO_2_ nanocomposites via the electrochemical deposition method. The fabrication process consisted of two main steps: in the first step, helical TiO_2_ nanotube arrays (NTAs) were prepared through anodizing a Ti foil. The second step was the electrodeposition of Cu and Cu_2_O nanoparticles on the TiO_2_ NTAs surface.

**Figure 4:**
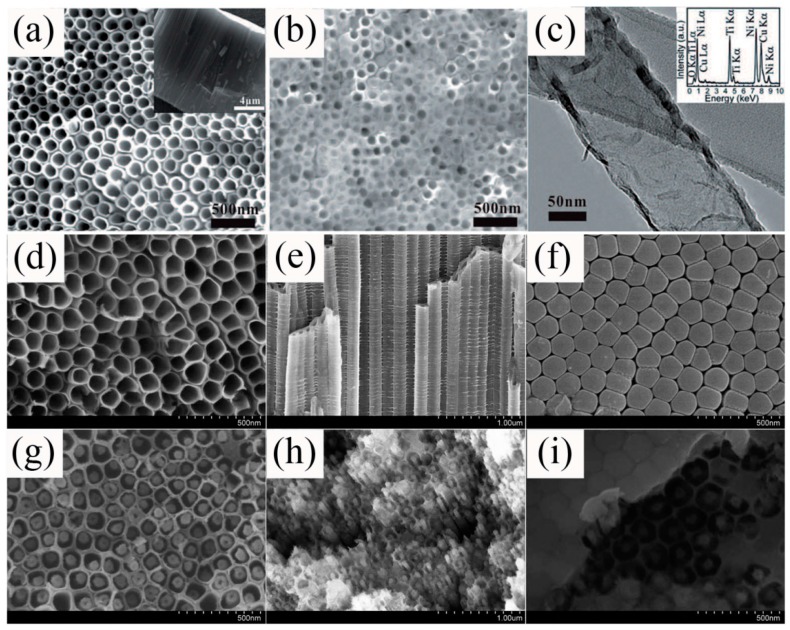
(**a**–**c**) SEM images of (**a**) TiO_2_ NTAs and (**b**) hydrothermally treated TiO_2_ NTAs; (**c**) TEM image of a typical NiTiO_3_/TiO_2_ NTs, the inset is the corresponding energy dispersive spectrometer (EDS) spectrum [79]. Copyright 2015 Wiley. (**d**–**i**) SEM images of as-anodized TiO_2_ NTs (**d**–**f**) and TiO_2_ NTs filled by Co-precursor nanorods (**g**–**i**) in top view(**d**,**g**), cross-sectional view (**e**,**h**), and bottom view (**f**,**i**) [80]. Copyright 2013 Royal Society of Chemistry.

Additionally, a great number of TiO_2_-based nanoheterostructures were prepared using electrochemical deposition combined with other synthesis processes, where 1D TiO_2_ arrays were commonly synthesized by electrochemical deposition, and then the as-prepared TiO_2_ arrays would be used as templates for heterostructure fabrication in the second synthesis step by other methods, including chemical deposition, hydrothermal/solvothermal methods, sol-gel method, and so on. For example, coaxial Ni/NiTiO_3_/TiO_2_ NTAs (Figure 4a–c) were synthesized by hydrothermally treating the as-anodized TiO_2_ NTAs [79]. Nano-coaxial p-Co_3_O_4_/n-TiO_2_ heterojunctions were synthesized first using electrochemical deposition and secondly using hydrothermal reaction [80]. The SEM images are shown in Figure 4d–i, where obviously the NTs with open top and closed bottom display high order and directionality. When soaking the as-anodized TiO_2_ NTs in Co-based solutions, new nanorods with smaller diameter can be formed in the center of the TiO_2_ NTs, representing the formation of nano-coaxial shaped of nanoheterostructures. Similarly, CdS/TiO_2_ nanoheterostructures were fabricated by electrochemical deposition and sequential chemical deposition [81]; Pd/TiO_2_ nanoheterostructures were synthesized by electrochemical deposition and a UV irradiation chemical method [82]; polypyrrole (PPy)/TiO_2_ heterojunctions were fabricated by chemical deposition and electronchemical deposition methods [78,83].

### 2.4. Chemical Deposition

**Figure 5:**
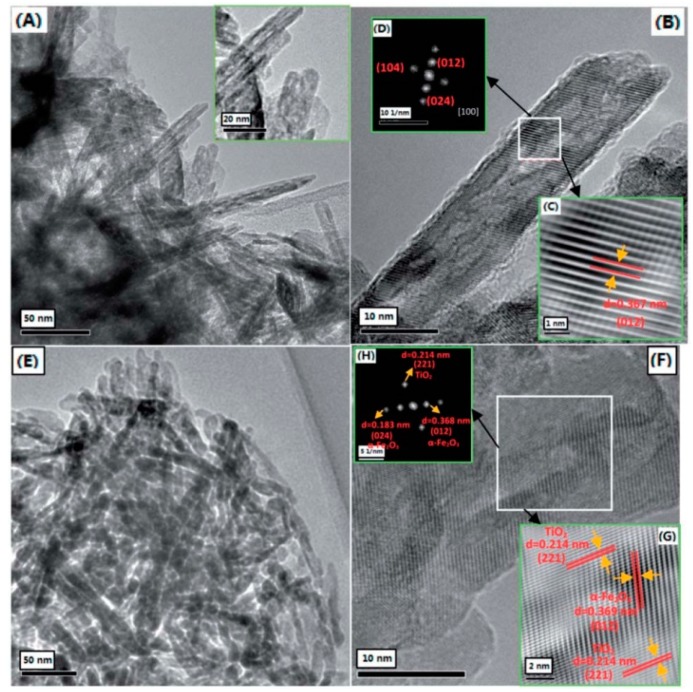
(**A**) TEM images of a-Fe_2_O_3_ nanorods; (**B**) HRTEM image of an individual a-Fe_2_O_3_ nanorod, the insets are the enlarged HRTEM image (**C**) and the corresponding fast Fourier transform (FFT) pattern (**D**) taken from the frame-marked region in (**B**); (**E**) TEM image of TiO_2_/a-Fe_2_O_3_ nanoheterostructures; (**F**) HRTEM image of TiO_2_/a-Fe_2_O_3_ nanoheterostructures, the insets are the enlarged HRTEM image (**G**) and the corresponding FFT pattern (**H**) taken from the frame-marked region in (**F**) [65]. Copyright 2014 Royal Society of Chemistry.

Chemical deposition is a convenient and low-cost technique for fabricating nanoheterostructures. For this method, the nanomaterials are obtained from the solid precipitation in the solution, and the concentration of the precursor, pH value, deposition temperature, and deposition time play important roles in the process. Nowadays, chemical deposition is generally applied in preparing either pure nanomaterials or composite nanomaterials. For example, brookite-TiO_2_/a-Fe_2_O_3_ nanoheterostructures have been synthesized via two-step facile chemical deposition without using any templates or surfactants [65], as shown in Figure 5.

Furthermore, chemical deposition combined with other methods can also be used to fabricate nanoheterostructures. Our group has recently reported the fabrication of Ag_2_O/TiO_2_/V_2_O_5_ nanoheterostructures (STV NHs) [84] through a two-step synthesis approach: the first step was the preparation of continuous TiO_2_/V_2_O_5_ nanofibers (TV NFs) using an electrospinning method, and the second step was the deposition of Ag_2_O nanoparticles on the TV NFs surfaces by the reaction of AgNO_3_ solution and NaOH solution. Based on this facile method, some other nanoheterostructures could be easily obtained, such as TiO_2_/CuO [85], TiO_2_/LiCl [86], and TiO_2_/FeOOH [87] nanoheterostructures.

It is worthy to note that room temperature chemical method is an efficient way to synthesize organic-functionalized TiO_2_ nanoheterostructures, such as TiO_2_/polypyrrole nanocomposites [88], TiO_2_-diltiazem/tetrachlorobismuth core-shell nanospheres (TiO_2_@DTMBi core-shell nanospheres) [89], polymer-functionalized TiO_2_ nanorods [90], molecularly imprinted polymer on TiO_2_ nanotubes (MIP@TiO_2_ NTs) [91], polypyrrole/TiO_2_ heterojunction (PPy/TiO_2_ heterojunction) [78], and Prussian blue/TiO_2_ nanowires (PB/TiO_2_ NWs) [63]. Typically, Wang and his co-workers [92] have reported a simple, low cost, and effective chemical method to fabricate polythiophene/Pd/TiO_2_ ternary composite nanoheterostructures, where TiO_2_ spheres were synthesized in water/acetone solvent, and then the Pd species were loaded on the TiO_2_ microspheres, finally the polythiophene was covered on the spheres to obtain polythiophene/Pd/TiO_2_ ternary composites.

### 2.5. Hydrothermal/Solvothermal Technique

**Figure 6:**
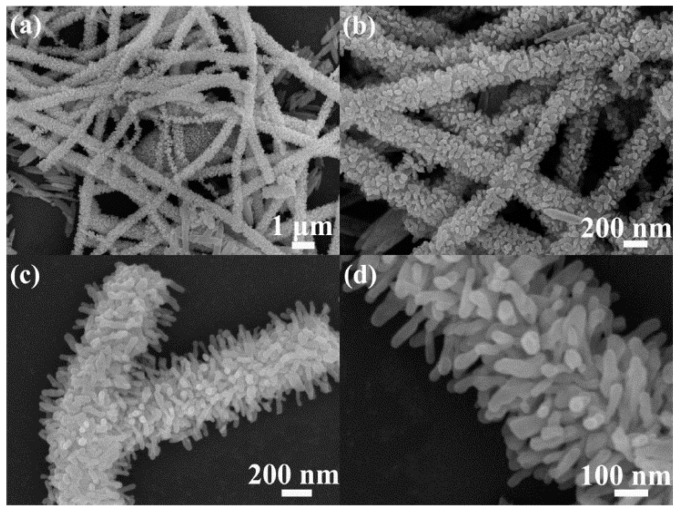
SEM images of branched *α*-Fe_2_O_3_/TiO_2_ nanoheterostructures: (**a**,**b**) panoramic and (**c**,**d**) magnified [62]. Copyright 2013 American Chemical Society.

Hydrothermal/solvothermal methods are commonly applied in the synthesis of powdery nanostructures. In the typical process, reagents (such as amines) and precursors are firstly mixed with each other in an appropriate ratio and then injected into a solvent, which can not only speed up the precursor dissolution but also accelerate the reaction between reagent and precursor. Finally the solution is added into a special hydrothermal synthesis reactor for the reaction of reagent and precursor and the growth of nanomaterials at relatively high temperature and high pressure. For example, TiO_2_/V_2_O_5_ nanoheterostructures could be prepared through solvothermally treating TiO_2_ nanoparticles in vanadium chloromethoxide precursor solution to growing V_2_O_5_ on TiO_2_ nanoparticle surfaces [93]. Similarly, branched 1D *α*-Fe_2_O_3_/TiO_2_ nanoheterostructures [62] were synthesized by growing *α*-Fe_2_O_3_ nanorods on the TiO_2_ nanofibers using hydrothermal treatment. Figure 6 displays the SEM images of the branched *α*-Fe_2_O_3_/TiO_2_ nanoheterostructures, it can be observed that the sample is mainly formed by branch-like nanofibers with loose and rough surfaces. In addition, one-dimensional carbon nanotube (CNT)-TiO_2_ heterostructures were prepared through a solvothermal route using multiwalled CNTs as templates [25]. As demonstrated by these examples, hydrothermal/solvothermal methods are suitable techniques to fabricate controlled nanoheterostructures.

### 2.6. Sol-Gel Method

The sol-gel method is a representative wet chemistry technique for synthesizing TiO_2_-based nanoheterostructures which involves relatively low growth temperatures and the morphology of the products can thus be controlled. The general process is as follows: first, prepare the sol gel, then heat the solution at high temperature combined with vigorous stirring to make it hydrolyze, finally carry out a condensation reaction to obtain nanomaterials. For instance, Lee et al. have fabricated a quartz crystal microbalance (QCZ) gas sensor based on the polyacrilic acid (PAA)/TiO_2_ nanofilm, where the (TiO_2_/PAA)*_n_* (*n* = 5, 10, and 20) nanofilms were deposited on gold-coated quartz crystal microbalance electrode using gas-phase surface sol-gel method [94].

### 2.7. Electrospinning

**Figure 7:**
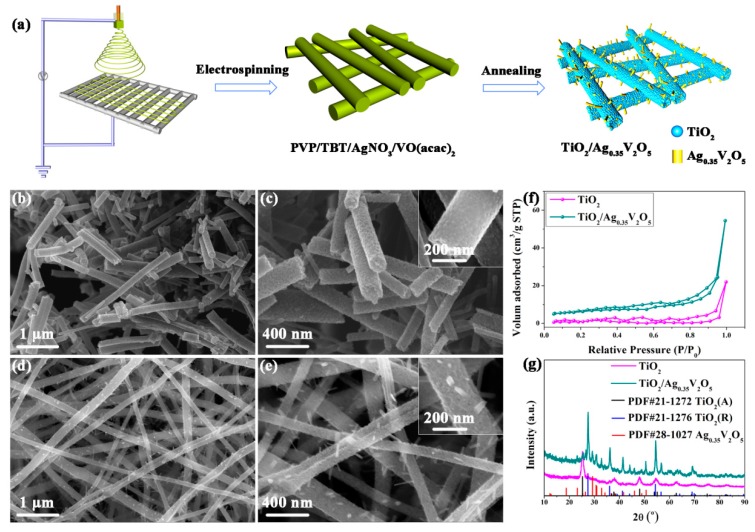
(**a**) A diagram of the electrospinning process; (**b**–**e**) SEM images of (**b**,**c**) TiO_2_ nanofibers and (**d**,**e**) TiO_2_/Ag_0.35_V_2_O_5_ branched nanoheterostructures; (**f**) N_2_ adsorption/desorption isotherms and (**g**) XRD patterns of TiO_2_ nanofibers and TiO_2_/Ag_0.35_V_2_O_5_ branched nanoheterostructures [95]. Copyright 2016 Nature.

Since the electrospinning technique was first reported, more and more nanomaterials have been prepared by researchers via this simple method. During the typical process, a glutinous precursor solution is injected through a thin spinneret and then is stretched to form ultralong nanofibers; finally annealing the as-prepared samples at appropriate temperature can produce very highly crystalline nanofibers. In recent years, our group has successfully synthesized TiO_2_/Ag_0.35_V_2_O_5_ branched nanoheterostructures using a simple one-step electrospinning method [95,96]. The synthesis process of TiO_2_/Ag_0.35_V_2_O_5_ nanoheterostructures and characterization of the nanoheterostructures are presented in Figure 7. 

In the typical electrospinning process, the consistence of the composite, working voltage, inner diameter of the spinneret, distance between the spinneret tip and the collector substrate, and annealing temperature can all affect the morphology of the nanomaterials. Furthermore, Ag/TiO_2_ and TiO_2_/V_2_O_5_ nanoheterostructures have also been prepared using the electrospinning process by our group [97,98,99]. What’s more, various type of TiO_2_-based nanoheterostructures fabricated by electrospinning process have been reported by other researchers, such as TiO_2_/In_2_O_3_ [61], LiCl/TiO_2_ [100], and Pt/TiO_2_ [101] nanoheterostructures. Especially, Du’s group has synthesized TiO_2_/ZnO core-sheath nanofibers using a coaxial electrospinning method [102].

## 3. Diverse Nanoheterostructural Gas Sensors

During the past decades, TiO_2_ has attracted considerable attention because it was regarded as a promising candidate for waste gas detection. Unfortunately, the poor sensing activity of high resistance n-type TiO_2_ seriously influences the development of TiO_2_-based gas sensors. Recently, various heterostructured sensing materials have been reported. The coupling of different materials can result in improved sensing activity. In this section, we will focus on the sensing performance of multifarious TiO_2_-based nanoheterostructure gas sensors, highlighting in particular, semiconductor/semiconductor nanoheterostructures.

### 3.1. Semiconductor/Semiconductor Nanoheterostructures

One of efficient ways to devise outstanding gas sensors is the modification of TiO_2_ by coupling with other semiconductors to form nanoheterostructures, which could display enhanced sensitivity and selectivity, faster response/recovery times, and/or lower operational temperatures than pure TiO_2_ [45,46,100,103,104]. Table 1 summarizes the different TiO_2_-based semiconductor/semiconductor nanoheterostructures and their performance in the gas sensing detection field. For example, Zeng et al. [105] have successfully fabricated a novel gas sensor based on the SnO_2_-TiO_2_ hybrid nanomaterials. 

**Figure 8:**
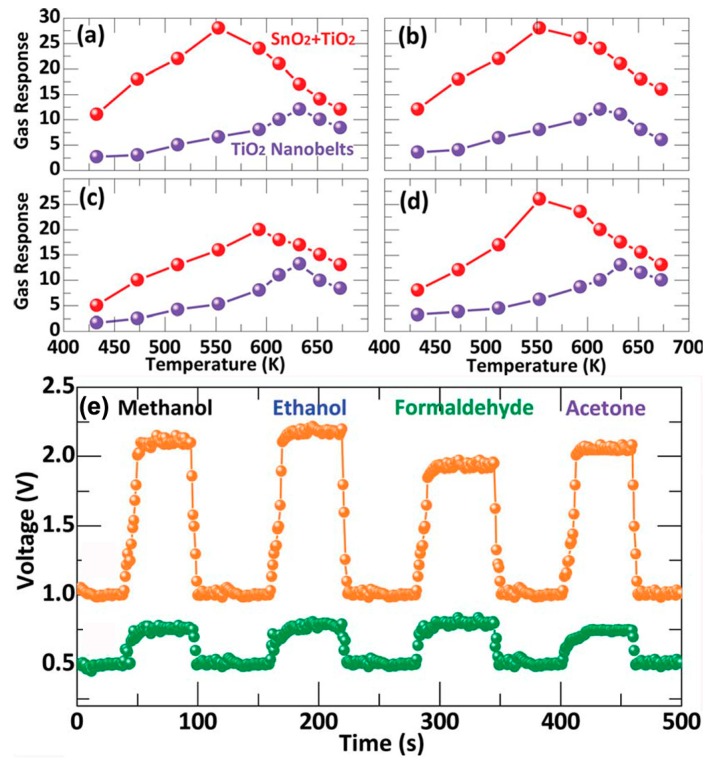
(**a**–**d**) Gas response of the TiO_2_ nanobelts and the SnO_2_-TiO_2_ hybrid oxides based sensors to 400 ppm methanol (**a**); ethanol (**b**); formaldehyde (**c**); and acetone (**d**) gases at different operating temperatures; (**e**) Response/recovery characteristics of the TiO_2_ nanobelts and the SnO_2_-TiO_2_ hybrid oxides based sensors operated at 593 K to 400 ppm methanol, ethanol, formaldehyde, and acetone [105]. Copyright 2012 Royal Society of Chemistry.

They demonstrated that the SnO_2_ nanospheres-functionalized TiO_2_ nanobelts-based sensor displayed very outstanding sensing properties, higher response and lower operating temperature, than pure TiO_2_ nanobelts-based sensors, as shown in Figure 8. 

**Figure 9:**
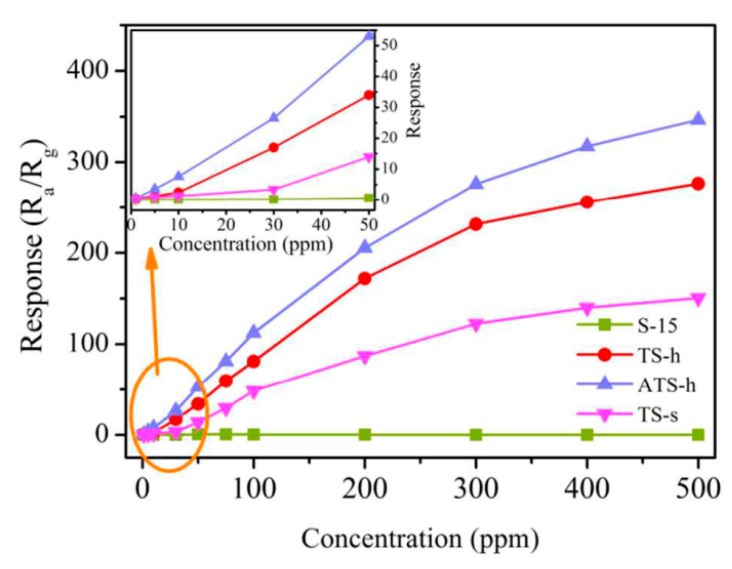
Gas responses of different sensors (S-15: SBA-15, TS-s: TiO_2_/SnO_2_ (soft template), TS-h: TiO_2_/SnO_2_ (hard template), ATS-h: Ag-(TiO_2_/SnO_2_)) to ethanol operated at 275 °C, the inset is the calibration curve within the concentration ranging from 1 ppm to 50 ppm [104]. Copyright 2016 Royal Society of Chemistry.

Tomer and Duhan [104] reported a mesoporous Ag-(TiO_2_/SnO_2_) structure which exhibited high sensitivity, a low detection limit (1 ppm), and wide detection range (1 ppm to 500 ppm) to ethanol, as shown in Figure 9. Moreover, the mesoporous Ag-(TiO_2_/SnO_2_) nanohybrid sensor also displayed excellent stability and high selectivity for ethanol.

#### 3.1.1. Sensing Mechanism

Although the mechanism of the heterostructures has not been investigated explicitly, it is clear that the enhanced sensing properties for semiconductor/semiconductor nanoheterostructures should be related to the heterojunction constracted at the interface between two semiconductors, where the changes of heterojunction energy barrier immerged into different gas atmospheres are benefit to the improvement of sensing properties.

**Figure 10:**
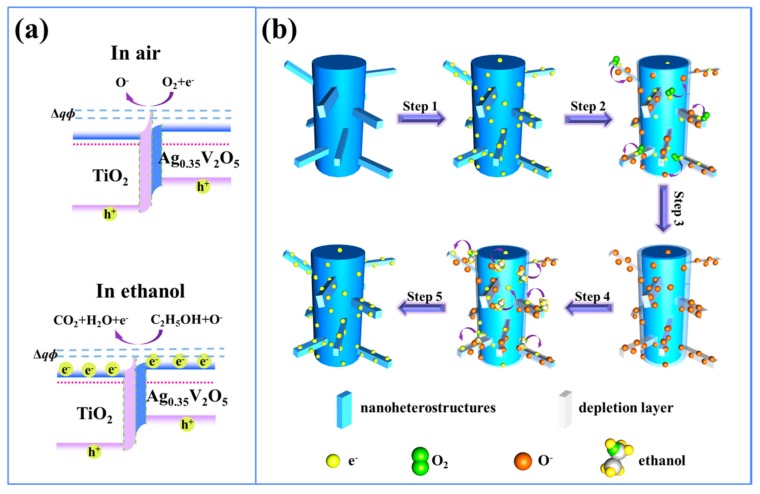
The proposed sensing mechanism diagram of TiO_2_/Ag_0.35_V_2_O_5_ nanoheterostructures. (**a**) Schematic band structure of TiO_2_/Ag_0.35_V_2_O_5_ heterojunction exposed in air and ethanol gases (*qΦ*: energy barrier); (**b**) Sensing model of the TiO_2_/Ag_0.35_V_2_O_5_ nanoheterostructured sensor in air (Steps 1–3) and in ethanol (Steps 4–5) [95]. Copyright 2016 Nature.

One possible explanation for the enhancement of these heterostructure-based sensors is the heterojunctions formed between TiO_2_ and other semiconductors. On the basis of band atructures and conductivity type of the semiconductors, two main types of semiconductor/semiconductor heteroatreuctures, n-n heterojunctions and p-n heterojunctions, can be considered. The work function (highest potential of valence band (VB)), electron affinity (lowest potential of CB), and bandgap of the coupled semiconductors determine the electron/hole dynamics in the heterojunctions. Since the work function of TiO_2_ is different from that of coupling semiconductors, the electrons will transfer from one semiconductor to another, thus resulting in an additional depletion layer and an energy barrier at the interfaces of two semiconductors. Compared with the pure semiconductors, the conductivity of heterojunctions is mainly detrermined by the energy barrier, and the relationship between resistance and energy barrier of the heterojunctions can be presented by the following equation:

(3) R∝B exp(qΦ/kT)

where *B* is a constant, *k* is the Boltzmann constant, *T* is the absolute temperature, and *qΦ* is the effective energy barrier at the heterojunction. When the heterostructures are in an air atmosphere, the electrons can be adsorbed by oxygen molecules to turn into various oxygen ions including O^−^, O^2−^, and O_2_^−^, accordingly the height of the energy barrier in heterojunctions will increase, as shown in the first figure of Figure 10a. Similarly, the energy barrier height will further increase when put into oxidizing gases, whereas in reducing gases, the gases can react with the oxygen ions and result in the release of adsorbed electrons, therefore the energy barrier height will decrease, as shown in the second figure of Figure 10a. According to Equation (3), R_a_/R_g_ is proportional to exp(Δ*qΦ*), thus the notable changes of energy barrier height can cause remarkable changes of the resistivity and superior enhancement of sensing properties for heterojunctions. For instance, Fe_2_O_3_/TiO_2_ tube-like nanoheterostructures [107] and TiO_2_/Ag_0.35_V_2_O_5_ branched nanoheterostructures [95] based sensors exhibited improved ethanol sensing performance compared with pure matrix sensors.

Additionally, a synergetic effect between different nanomaterials is also regarded as one of important reasons for the enhanced sensing properties of nanoheterostructures. In fact, the synergetic effect is related only to the situation where both of the pure materials exhibit high sensitivity to the tested gases [61].

It is well known that some metal oxide semiconductors are effective catalysts which can help decompose organic gases. Therefore, the catalytic effect of TiO_2_ and other materials should be taken into account in the sensing performance enhancement of nanoheterostructures. For example, according to the previous reports by our group [98,131], the TiO_2_/V_2_O_5_ nanoheterostructures can act as much more effective catalysts than pure TiO_2_ nanofibers, and should be capable of promoting the sensing reaction between volatile organic solvents (VOCs) and oxygen ions adsorbed at the surface, thus a TiO_2_/V_2_O_5_ nanoheterostructures-based ethanol sensor displays much higher sensitivity than a pure TiO_2_-based sensor [97].

In addition to the heterojunction effect, synergetic effect, and catalytic effect, there may be other mechanisms in play for enhancing the sensing performance of nanoheterostructure. For example, Chen et al. [108] considered that the electron transfer are facilitated because of the formation of SnO_2_/TiO_2_ heterostructures, thus the gas sensing response including sensitivity and selectivity is efficiently enhanced as a result of increased charge carrier concentration.

**Figure 11:**
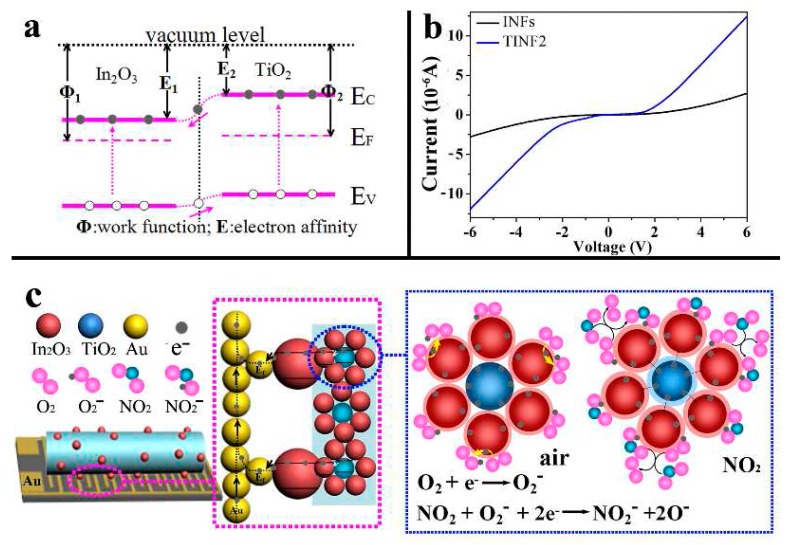
(**a**) Energy band diagram of In_2_O_3_ and TiO_2_, E_C_: conduction band, E_V_: valence band; (**b**) I-V curves of In_2_O_3_ nanofibers (INFs) and In_2_O_3_ beads@TiO_2_-In_2_O_3_ composite nanofibers (TINF2) thin film sensors in air at room temperature (the gate voltage Vg = 0.1); (**c**) The gas sensing reactions based on Schottky junction between Au electrode and In_2_O_3_ beads [61]. Copyright 2015 American Chemical Society.

Actually, there is more than one reason for the enhanced mechanisms of nanoheterostructure sensors. Park and coworkers [64] demonstrated that the improved ethanol sensing performance of TiO_2_-core/ZnO-shell nanorods compared with that of pure TiO_2_ nanorods might be due to more efficient catalytic activity of ZnO and the potential barriers built in the heterojunctions. Wang and coworkers [61] found that the improved gas sensing activity of the porous single crystal In_2_O_3_ beads@TiO_2_-In_2_O_3_ composite nanofibers (TINFs) could be ascribed to the Schottky junction formed between single crystal In_2_O_3_ beads and the Au electrode, the increased carrier density derived from the TiO_2_ electron-donor, and the best gas absorption conditions provided by the surface-related defects, as shown in Figure 11.

#### 3.1.2. The Influence of Morphology on Sensing Performance

It is reasonable that the influence of nanoheterostructures morphology on sensing performance should be taken into account, where the gas sensitivity and the response/recovery time affected by gas absorption and gas diffusion can be improved by the large specific surface area and the especial morphology of materials. On the one hand, like for resistance-type gas sensors, the fundamental sensing process is the reaction of the target gases and the electrons at the surface of the sensing materials. Larger surface area means more surface active sites can be provided for gas absorption and reactions, resulting in more noticeable changes of resistance in different gases, consequently the sensitivity of the sensors can be improved. On the other hand, the electron exchange process can only occur within a thin layer, the width of this surface layer is determined by the Debye length (*L_D_*) of the materials, which is defined by following equation:

(4) $$L_{D}={\left(kT\varepsilon \varepsilon_{0}/q^{2}n_{c}\right)}^{1/2}$$

where *T* is the absolute temperature in Kelvin, *k* is Boltzmann’s constant, *ε*_0_ is the permittivity of vacuum, *ε* is the static relative dielectric constant, *n_c_* is the carrier concentration, and *q* is the electrical charge of the carrier. When *L_D_* is less or equivalent to the thickness of the nanostructures, the electrons in semiconductors can be totally depleted by the oxygen molecules adsorbed on the surface, thus will result in more evident resistance change after exposure in gases compared with that of not entirely depleted ones.

**Figure 12:**
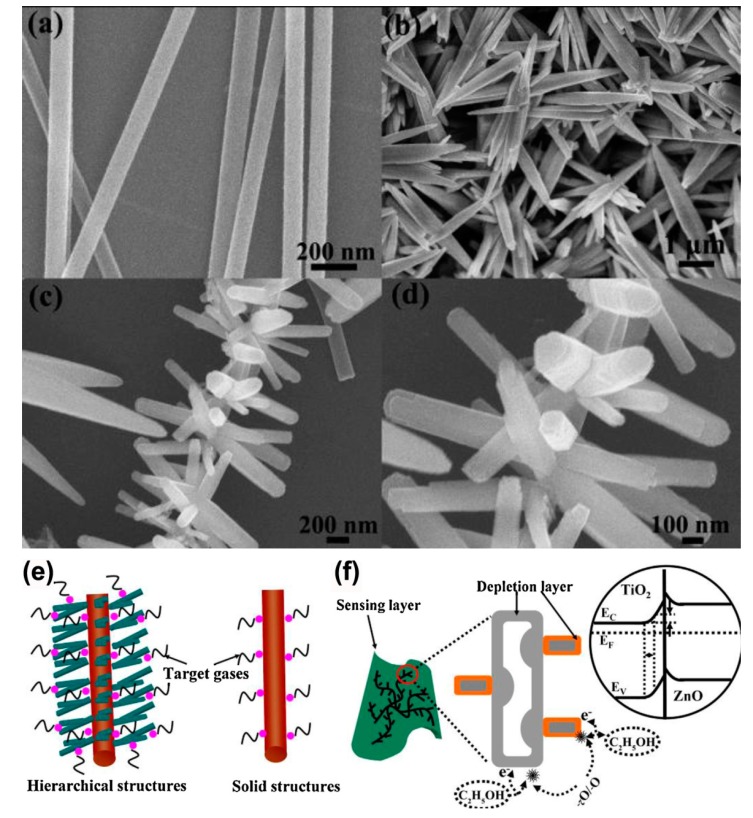
(**a**–**d**) SEM images of TiO_2_ nanofibers (**a**); ZnO nanorods (**b**); and ZnO-TiO_2_ nanoheterostructures (**c**,**d**); (**e**,**f**) Schematic diagram of catalytic reactions (**e**) and ideal band structure (**f**) of ZnO-TiO_2_ nanoheterostructures [110]. Copyright 2013 Elsevier.

Hierarchical nanostructures are a particularly promising choice for further enhancing the sensing performance because of their extremely large specific surface area and/or thin thickness (less or equivalent to *L_D_*). As a matter of fact, several hierarchical TiO_2_-based nanoheterostructures, such as branch-like *α*-Fe_2_O_3_/TiO_2_ hierarchical heterostructure [62], hierarchically assembled ZnO nanorods on TiO_2_ nanobelts [114], SnO_2_ nanospheres functionalized TiO_2_ nanobelts [105], have been fabricated into gas sensors. In particular, Zhu and coworkers have reported that *β*-FeOOH/TiO_2_ hierarchical heterostructures exhibited remarkably high sensitivity and reversibility [87]. Our group [95] demonstrated that the enhancement of the TiO_2_/Ag_0.35_V_2_O_5_ gas sensor could be attributed to the extraordinary branched-nanofiber structures with large surface area and thin branch diameter (the semidiameter of the branches was equivalent to the depletion layer of the Ag_0.35_V_2_O_5_), where more gas molecules could be absorbed and electrons in the Ag_0.35_V_2_O_5_ nanobranches could be totally depleted by the oxygen molecules adsorbed on the surface (Figure 10b). Furthermore, Deng et al. [110] have found that the enhanced performance of the ZnO-TiO_2_-based gas sensors could be ascribed to the hierarchical structures, as shown in Figure 12, where the high specific surface area could result in a large number of gas molecules absorbed on the surface of ZnO-TiO_2_ nanoheterostructures when compared with the pure TiO_2_ nanofibers, additionally, well aligned structures of the nanoheterostructures would cause the unhindered gas diffusion to the whole surface of the sensor.

**Figure 13:**
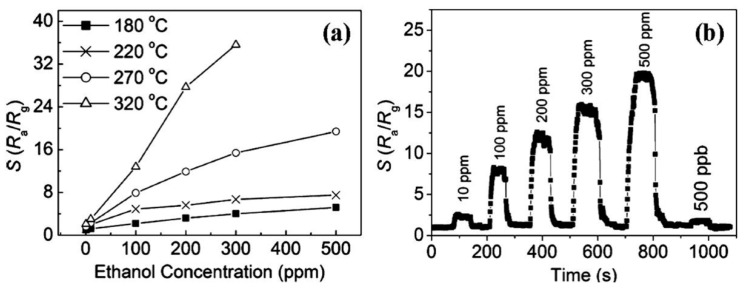
(**a**) Sensing response of the Fe_2_O_3_/TiO_2_ tube like nanoheterostructures to ethanol at different temperatures; (**b**) Time dependent sensing response of the Fe_2_O_3_/TiO_2_ tube like nanoheterostructures to ethanol vapor at 270 °C [107]. Copyright 2012 American Chemical Society.

**Figure 14:**
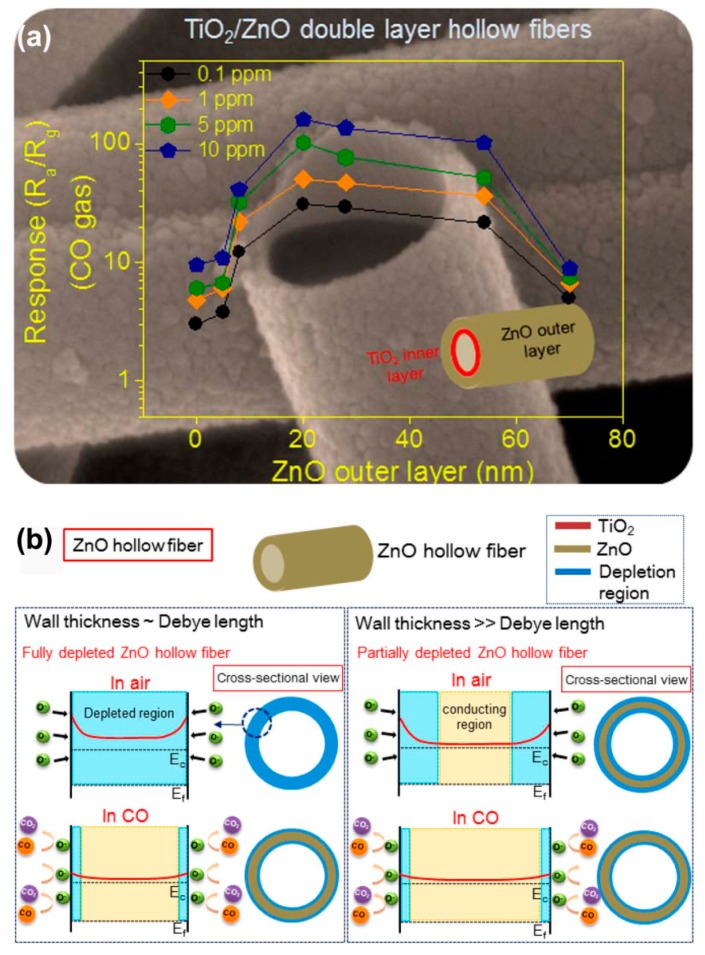

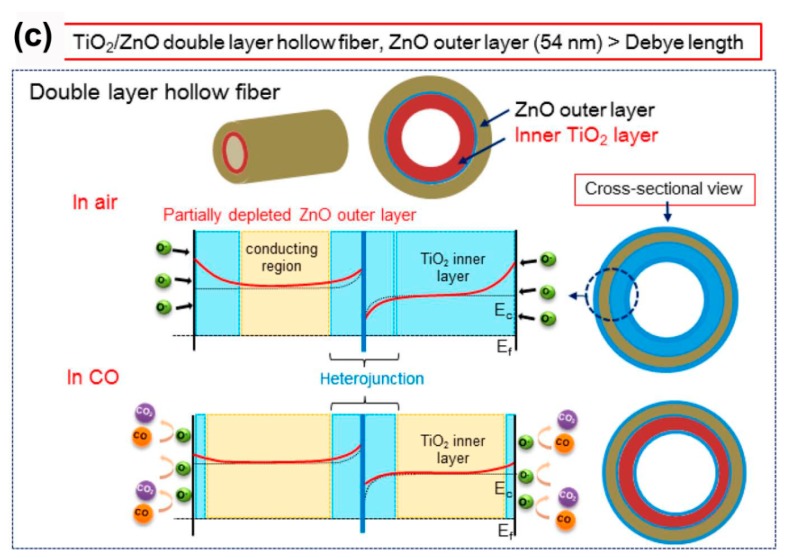
(**a**) Sensing response of TiO_2_/ZnO double layer hollow fibers to CO gas as a function of ZnO outer layer thickness; (**b**,**c**) Schematic diagrams of sensing mechanism of (**b**) ZnO hollow fibers and (**c**) TiO_2_/ZnO double-layer hollow fibers [72]. Copyright 2014 American Chemical Society.

In addition to island type hierarchical heterostructured sensors, core/shell type and hollow type nanoheterostructures have also attracted much attention in gas sensor research. Recently, Zhu et al. [107] synthesized tube-like Fe_2_O_3_/TiO_2_ core/shell nanoheterostructures (Figure 13). The special core/shell nanoheterostructures could act as a superior ethanol sensor material with respect to the pristine one, as shown in Figure 13. Katoch and coworkers [72] demonstrated that TiO_2_/ZnO double layer hollow fibers exhibited superior CO sensing performance compared to the ZnO single layer hollow fibers, as shown in Figure 14a. The enhancement was ascribed to the fact that the electrons in ZnO outer layer could be easily absorbed to TiO_2_ inner layer, thus ZnO would become more resistive owing to the noticeable loss of electrons, as shown in Figure 14b,c. When exposed in CO gas atmosphere, the resistance of ZnO outer layer would partially regain its original value, this could lead to more noticeable resistance change for the TiO_2_/ZnO double layer hollow fibers in detecting CO gas.

### 3.2. Carbon-Group-Materials/Semiconductor Nanoheterostructures

The formation of carbon-group-materials/semiconductor heterostructures is also an important technique to improve the gas sensing performance of TiO_2_. In recent years, carbon-group-materials have attracted much attention for applications in gas sensors [1,12,15,132]. The nanostructured carbon materials including carbon nanotubes (CNTs) and graphene possess outstanding physical, chemical and electrical properties, such as good flexibility, large surface area, high chemical stability, and high electrical conductivity [14,133]. These excellent features make them extremely suitable for use as candidates in enhancing semiconductors’ sensing properties [132,134,135,136]. It has been demonstrated that the absorptivity, conductivity, and/or electrochemical reaction of some small gas molecules of carbon/TiO_2_ nanoheterostructures could be promoted, thus the nano- heterostructures can display remarkably improved sensing performance [75,137,138,139]. Table 2 summarizes the carbon/TiO_2_ nanoheterostructures and their performance in the detection of gases.

The mechanism of enhanced gas sensing performance of the carbon/TiO_2_ nanoheterostructures is proposed to occur as follows: first, carbon-group-materials possess huge specific surface areas and nanoscale structures, thus a large number of surface sites are exposed for reacting with gases, therefore various gases can be easily detected at lower operating temperatures [140,141]. Second, the electric conductivity of carbon-group-materials is much higher in comparison with TiO_2_, this can reduce the resistance and enhance the electrons transport capability of heterostructured sensors, thus making the nanoheterostructures based sensors work at lower operating temperature [142,143]. Third, since TiO_2_ displays n-type semiconductor characteristic and carbon group- materials (graphene, CNTs) display p-type semiconductor characteristic, hence, a competitive mechanism may occur surrounding the carbon-group-materials/TiO_2_ heterojunctions, which can lead to enhanced gas sensitivity owing to the decrease of the work function (barrier height) or increase of the conductivity of TiO_2_ sensitive layer [144,145]. Furthermore, the catalytic activity of TiO_2_ should also be considered as one of important reasons for the improved sensing performance of carbon-group-materials/TiO_2_ nanoheterostructures [146,147].

As an emerging carbon material, graphene is regarded as a promising candidate for application in gas sensors due to its huge surface area, high electrical conductivity, inherently low electrical noise, environmental ultra-sensitivity, and ease microfabrication. In particular, the combination of graphene and TiO_2_ can achieve efficiently improved sensing performance. 

**Figure 15:**
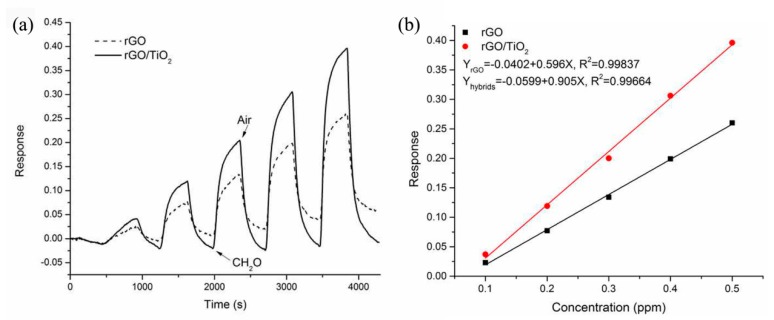
(**a**) response curves and (**b**) response values of pure rGO and rGO/TiO_2_ layered films to 0.1–0.5 ppm CH_2_O [75]. Copyright 2015 Elsevier.

In Ye’s research [75], rGO/TiO_2_ layered thin film were prepared on the interdigital electrode substrates via a spray method. Formaldehyde sensing tests demonstrated that the layered thin film exhibited high sensitivity (0.905 ppm^−1^), and a reversible and linear response to 0.1–0.5 ppm formaldehyde at room temperature, and that this could be ascribed to the positive synergetic effect of the two materials, as shown in Figure 15. Xiang and coworkers [146] developed a room-temperature sensitive NH_3_ gas sensor using TiO_2_@PPy-graphene nanocomposites. The sensor displayed high sensitivity (102.2%), superior reproducibility, and excellent selectivity to 50 ppm NH_3_. Additionally, Wang et al. [147] synthesized TiO_2_/graphene composite film for O_2_ sensing upon exposure to UV light. They found that the outstanding sensitivity of the composite film could be ascribed to the synergetic effect of the ultrasensitivity of single-layer graphene to the environment and photocatalytic activity of TiO_2_.

**Figure 16:**
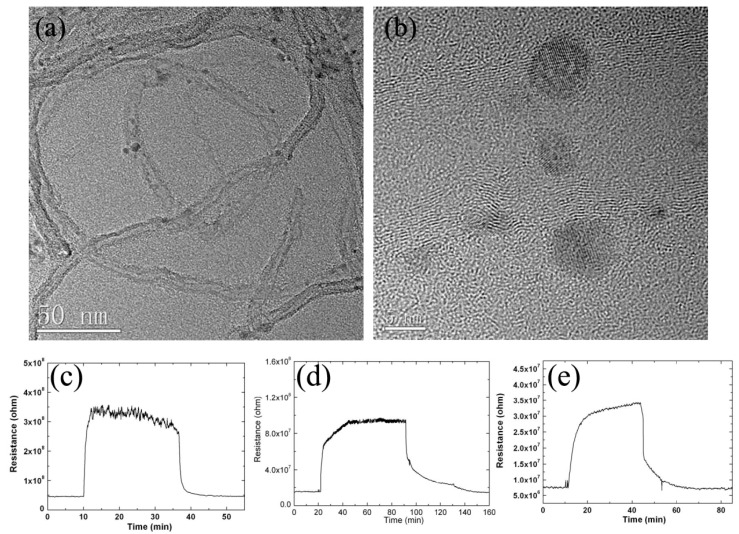
(**a**,**b**) HRTEM images of CNT/TiO_2_ nanocmposites; (**c**–**e**) Response to 10 ppm of O_2_ in CO_2_ flow at 450 °C for (**a**) a TiO_2_/MWCNT sensor annealed at 500 °C; (**b**) a TiO_2_/MWCNT sensor annealed at 600 °C; and (**c**) a Nb-doped TiO_2_/MWCNT sensor annealed at 500 °C [154]. Copyright 2008 Institute of Physics.

**Figure 17:**
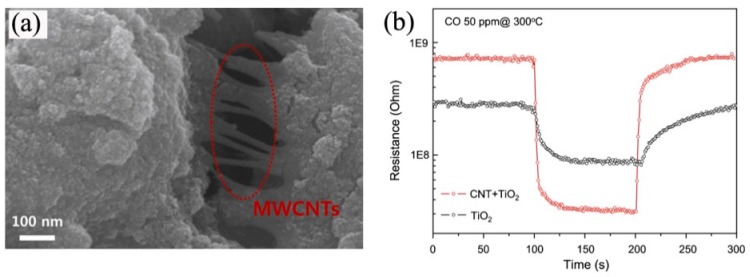
(**a**) SEM image of MWCNTs/TiO_2_ xerogel film; (**b**) CO sensing properties of pure TiO_2_ xerogel film and MWCNTs/TiO_2_ xerogel film to 50 ppm CO at 350 °C [151]. Copyright 2013 Elsevier.

Like graphene, CNTs have also been widely studied as preeminent gas sensing materials. Recently, multifarious effort has been devoted to the design and fabrication of CNTs/metal oxide nanocomposites [138,139,155,149]. The new composite materials will maintain the original properties of each component, or even show a synergistic effect, which is exceedingly significate to sensing performance. For example, Llobet et al. [154] proposed an effective O_2_ sensor based on CNTs/TiO_2_ hybrid films. The researchers compared the sensing properties of CNTs/TiO_2_ hybrid film and Nb-doped TiO_2_ films, and the results showed that the sensitivity of former was four times higher than that of latter, as shown in Figure 16. The outstanding sensing performance of CNTs/TiO_2_ hybrid films arose from their very large surface area because of the central hollow cores and outside walls of CNTs. Luca and coworkers [149] have developed a room temperature sensor using Pt/TiO_2_/MWCNTs composites. The results showed that increased resistance in response to H_2_ possibly indicated a p-type conduction mechanism of the composite sensing material. Lee et al. [151] have demonstrated that the MWCNTs/TiO_2_ xerogel composites film possessed better sensing performance. Compared with pure TiO_2_ xerogel, the nanocomposites displayed improved sensitivity (15.8), and lower response/recovery times (4/16 s), as shown in Figure 17. The improved sensing properties could be attributed to the increased surface-to-volume area and enhanced conductivity which were benefit from the p-n heterojunction formed at the interface of MWCNTs and TiO_2_. This result could also be confirmed by Kim et al. [152] who prepared a MWCNTs-modified direct-patternable TiO_2_ thin film based CO gas sensor. It was found that the incorporation of MWCNTs could induce increase of surface morphology and roughness and thus resulted in promoted sensing performance.

### 3.3. Organic/Inorgnic Nanoheterostructures

Nowadays, more and more requirements for gas sensors applied in the safety control and environmental monitoring have been put forward. In these efforts, designing and manufacturing suitable and efficient sensing materials is an important factor in obtaining highly efficient sensors [156]. In recent years, conductive polymers were considered a promising sensing element because of the significant changes in electrical and optical properties of such materials exposed in different gas atmospheres. In particular, the simple preparation and high sensing performance at room temperature of these polymers make them more acceptable for applications in many fields [157].

Conducting polymer-based nanocomposites composed of metal oxides nanomaterials and conducting polymers have been well developed, and the corresponding sensors exhibit great potential in probing various hazardous gases [91] due to their enhanced sensing properties, such as fast and reversible responses to target gases at room temperature [92], and more noticeably, that defined organic material modified semiconductor sensors exhibit outstanding sensing selectivity to a single gas species because of their exclusive chemical and electronic conditions [6,158,159,160]. Particularly, organic-functionalized TiO_2_ nanoheterostructures also exhibit significantly improved characteristics in gas sensing compared with pure TiO_2_ nanomaterials [83,91,94,161,162] and thus have been investigated by several research groups, as shown in Table 3.

Till now, a number of literatures proposed the sensing mechanism of polymer/TiO_2_ nanoheterostructures. One of generally accepted factor is that the nanostructures of the heterostructures are beneficial to the high sensing properties, which mainly result from their large specific surface areas and much more active sites for the adsorption and interactions with the gases [163,164,165]. What is more, the formation of heterojunction at the interface between TiO_2_ and polymers is usually regarded as a main factor for the ultrahigh sensitivity of the nanoheterostructures [83]. At the heterostructure interface, the electrons will transfer from the material with higher Fermi level to another with lower Fermi level, while the holes will transfer rightabout until the Fermi levels is equalized. In this process, electron depletion layer, an efficient current switch, will form at the interface of the heterostructures, thus leading to high gas sensitivity [166,167,168].

**Figure 18:**
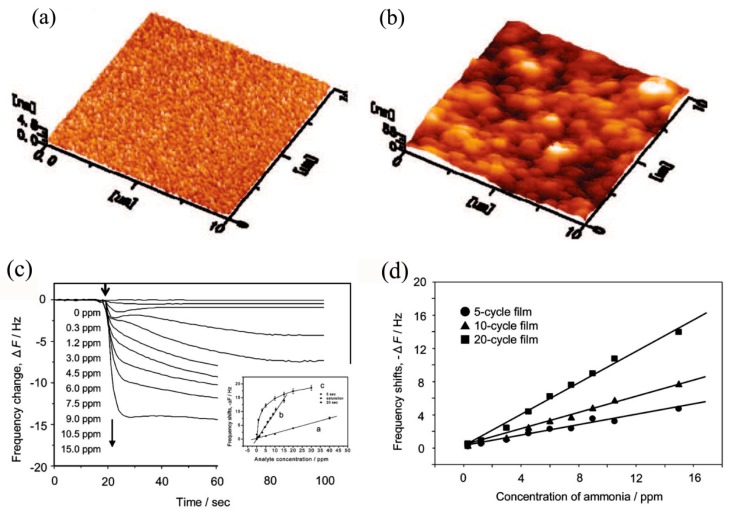
(**a**,**b**) Atomic force microscope (AFM) images of the surface morphology of PAA25 (**a**) and PAA400 (**b**) deposited on TiO_2_ gel-immobilized mica; (**c**) Dynamic responses of the quartz crystal microbalance (QCM) electrode coated with a (TiO_2_/PAA400)_20_ film to ammonia at different concentrations. The inset shows a comparison of the calibration curves with data taken at different times; (**d**) Calibration curves for (TiO_2_/PAA)*_n_* (*n* = 5, 10, and 20) films [94]. Copyright 2010 American Chemical Society.

**Figure 19:**
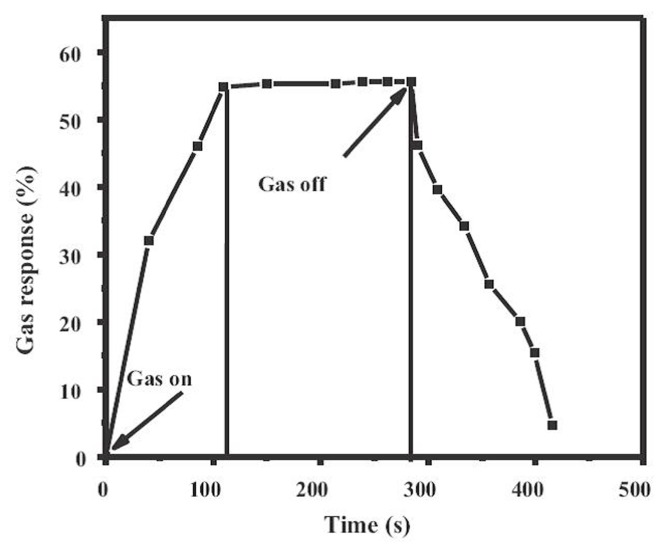
Gas response of a PPy/TiO_2_ heterojunction at a fixed voltage of +0.6 V at concentration of 1040 ppm of LPG [83]. Copyright 2013 Elsevier.

Polyacrylic acid (PAA) is one of the appropriate choices for probing NH_3_ gas because the free carboxylic functional groups present on the PAA surface possess high sensitivity and selectivity to NH_3_ molecules [174]. The incorporation of PAA and metal oxide nanomaterials may solve the long recovery time at higher NH_3_ concentration (>1 ppm) and undesired sensitivity to humidity of the existing inorganic semiconductor sensors [175]. For instance, Lee and coworkers [94] have demonstrated that TiO_2_/PAA-based amine gas sensors exhibited fast and stable response in a wide relative humidity range of 30%–70%, furthermore, a good linear response was also observed in the NH_3_ concentration range between 0.3 ppm and 15 ppm, as shown in Figure 18. One of possible reasons for the decreased influence of humidity on gas sensitivity was that the presence of the TiO_2_ would suppress the mobility of PAA, therefore the influence of water molecules on the sensing response would be reduced. As another very interesting conducting polymer, polypyrrole (PPy) has been widely investigated due to its relatively good environmental stability and easily controlled surface carrier properties adjusted by altering the dopant species in PPy during the synthetic process [169]. Accordingly, a number of researchers have attempted to enhance the gas sensing performance of TiO_2_ by introducing PPy to form organic/inorgnic nanoheterostructures. For example, Bulakhe and coworkers [83] have prepared a PPy/TiO_2_ heterojunction-based liquefied petroleum gas (LPG) sensor which could operate at room temperature. The maximum sensing sensitivity of 55% to 1040 ppm LPG was observed, as shown in Figure 19. Compared with other room temperature LPG sensors, the PPy/TiO_2_ heterojunction-based sensor could work at low LPG concentrations and showed promoted response/recovery times (112/131 s), indicating the PPy/TiO_2_ heterojunction was a promising choice for a room temperature LPG sensor. Furthermore, Tai et al. [88] have investigated the NH_3_ sensing performance of TiO_2_/PPy nanocomposite ultrathin films. The results revealed that the TiO_2_/PPy ultrathin film presented outstanding sensing performance, such as shorter response/recovery time comparing with pure PPy thin film based sensor. Additionally, in the work of Wu et al. [169], the PPy/TiO_2_ composite thin film based sensor displayed much lower detection limit of 2 ppm to NH_3_ gas. The improvement of sensing performance of PPy/TiO_2_ heterostructures is mainly attributed to the formation of p-n junctions at the interface between TiO_2_ and organic PPy.

**Figure 20:**
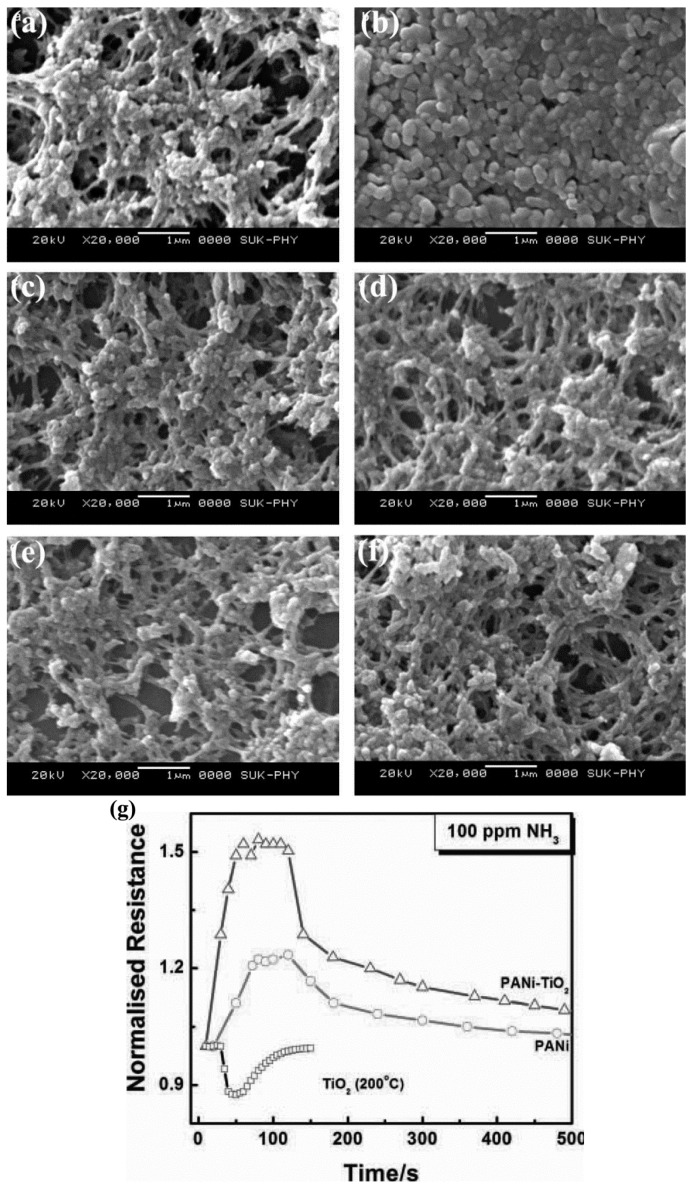
(a–f) SEM images of PANi (a); TiO_2_ (b); and PANi–TiO_2_ (20–50 wt %) films (c–f); (g) Response of the pure TiO_2_, pure PANi, and nanocomposite of the PANi-TiO_2_ film toward 100 ppm NH_3_ gas at room temperature [74]. Copyright 2012 Wiley.

Moreover, polyaniline (PANi) has attracted much attention in commercial applications due to its excellent environment stability, novel photoelectrical and electrical characteristics, and easy fabrication [170]. It has been demonstrated that combining TiO_2_ with PANi can enhance the sensitivity, selectivity, and stability of the resulting sensors [88]. For example, Gong et al. [49] have reported an ultrasensitive NH_3_ gas sensor based on PANi/TiO_2_ fibers p-n heterojunctions, it was found that the p-n heterojunctions could act as electronic transmission switches when NH_3_ gas was absorbed by PANi, thus resulting in the enhancement of sensing properties. Similarly, using TiO_2_ nanoparticles modified PANi fibers as sensor, a fast response of 2–3 s and a low detection limit of 1 ppm at room temperature were obtained by Tai et al. [88]. Additionally, Pawar et al. [74] have also investigated the sensing performance of nanostructured PANi/TiO_2_ films synthesized via a spin-coating method on glass substrates, the results revealed that the nanocomposites film exhibited much enhanced sensing performance, such as higher sensitivity and lower respons/recover time toward NH_3_ at room temperature, as shown in Figure 20.

## 4. Conclusions

In the last few decades, continuous breakthroughs in the fabrication, modification and application of TiO_2_-based gas sensors have been reported. In this review, we describe the universal tactics and new progress in the preparation of TiO_2_-based nanoheterostructures for exhaust gases detecting. We focus on then synthetic methods and sensing performances of TiO_2_-based nanoheterostructures, including semiconductor/semiconductor nanoheterostructures, noble metal/semiconductor nanoheterostructures, carbon-group-materials/semiconductor nanoheterostructures, and organic/inorganic nanoheterostructures, which are summarized as follows:

(1) Coupling TiO_2_ by with other semiconducting materials to form heterostructures could result in enhanced sensitivity and selectivity, faster response/recovery times, and/or lower operational temperatures than pure TiO_2_. The enhanced sensing properties could be related to the heterojunctions formed at the interface between two semiconductors, the synergetic effect and catalytic effect. Additionally, the influence of the nanoheterostructures’ morphology on sensing performance should also be taken into account, where the gas sensitivity and the response/recovery time affected by gas absorption and gas diffusion can be promoted by the specific surface area and the special morphology of structures. As compared with other types of nanoheterostructure-based sensors, the minimization of detectable levels of the semiconductor/semiconductor nanoheterostructure-based sensors might not bear comparison with the carbon-group-materials/semiconductor nanoheterostructures, and their operation temperatures might be higher than that of noble metal/semiconductor nanoheterostructures and organic/inorganic nanoheterostructures, however, their most excellent stability make the semiconductor/semiconductor nanoheterostructures more acceptable when applied in many fields.
(2) Combining carbon-group-materials with TiO_2_ is considered an efficient way to improve the gas sensing performance of TiO_2_. It has been demonstrated that the absorptivity, conductivity, and/or electrochemical reactions of some small gas molecules with carbon/TiO_2_ nano- heterostructures could be promoted, thus the nanoheterostructures can display remarkably improved sensing performance. Remarkably, carbon-group-material/semiconductor nano- heterostructure-based sensors display the most outstanding minimum detectable levels as compared with other nanoheterostructures, which could be attributed to their large surface area and the high electrical conductivity of the carbon-group-materials.
(3) Conducting polymer-functionalized TiO_2_ nanoheterostructures have also been demonstrated to be some of most promising materials for gas detection. These nanoheterostructures possess fast and reversible responses at room temperature due to the formation of organic/inorganic heterojunctions. It is noticeable that defined organic material modified semiconductor sensors exhibit outstanding sensing selectivity to a single gas species because of their exclusive chemical and electronic conditions. As a type of typical room temperature sensor, the controllable sensing selectivity of organic/inorganic nanoheterostructures is an outstanding characteristic that other types of nanoheterostructures do not possess.

In summary, much progress has been achieved in investigation of TiO_2_-based nanoheterostructured sensors, and they are widely applicable in the detection of various gases such as H_2_, CO, NH_3_, H_2_O, VOCs, etc. However, there are still some challenges in designing high-performance nanoheterostructured gas sensors. First, although the TiO_2_-based nano- heterostructured gas sensors can respond to various gases based on the resulting resistance changes, a single sensor cannot discriminate different analytes, and the response is susceptible to other gases. Thus, most current studies are focused on the quantitative analysis of target gases using only simple matrices rather than complex matrices and actual samples. To meet the needs of practical applications, one possible solution is combing multiple sensors in a multimodal module sensor system, which can display distinctive response patterns across the array in probing different actual samples. Second, the understanding to the electrons transport and response on the interfaces of nanoheterostructures is not detailed enough, which is critical for the design and optimization of highly efficient sensors. Therefore it is necessary to further elucidate the dynamic behavior of electrons at the interface and surface of nanoheterostructures so that researchers can design TiO_2_ based nanoheterostructures more rationally. Furthermore, although a variety of synthetic methods have been successfully used for preparing TiO_2_-based nanoheterostructured sensors in the laboratory conditions, but these are far away from the large quantity industrial production. In addition, the chemical stability of TiO_2_ based nanoheterostructures is still poor, which seriously hampers the performance and wide application of the sensors. Therefore, the in-depth understanding of the sensing mechanism and the establishing of general and reasonable design guidelines in TiO_2_ based nanoheterostructured sensors are significant to achieve substantial breakthroughs in the practical application of the sensors.

## Figures and Tables

**Table 1:** Summary of the gas sensing properties of TiO_2_-based semiconductor/semiconductor nanoheterostructure sensors.

TiO_2_-Based Nanoheterostructures	Fabrication Method	Size	Detection Gas	Detection Range	Response				Ref.
					Operation Temperature (°C)	Sensitivity	Response/Recovery Time	Concentration	
TiO_2_/Co_3_O_4_ acicular nanowires	hydrothermal + pulsed laser deposition	length: 1–3 μm; diameter: 200 nm	ethanol	10–500 ppm	160	R_g_/R_a_: 65		100 ppm	[106]
nano-coaxial p-Co_3_O_4_/n-TiO_2_ heterojunction	electronchemical anodization + hydrothermal process	nanotubes diameter: ~150 nm; core nanorods diameter: ~50 nm	ethanol		260	R_a_/R_g_: 40	1.4/7.2 s	100 ppm	[80]
Fe_2_O_3_/TiO_2_ tube-like nanostructures	hydrothermal + chemical deposition	diameter: 120 nm; length: 400 nm; outer wall thickness: 23.5 nm	ethanol	0.5–500 ppm	270	R_a_/R_g_: 19.4		500 ppm	[107]
SnO_2_-coated TiO_2_ nanobelts	hydrothermal	TiO_2_ nanobelts: length: over ten micrometers; width: 100–200 nm; thickness: 20–40 nm; SnO_2_ nanoplates: length: 20–200 nm; thickness: 20 nm	ethanol	10–500 ppm	43	R_a_/R_g_: 11.2	40/5 min	10 ppm	[108]
TiO_2_/SnO_2_ core shell nanocomposites	hydrothermal treatment + chemical deposition		ethanol	500–5000 ppm	200	R_a_/R_g_: 12.7	≤50/50 s	1000 ppm	[109]
SnO_2_ nanospheres functionalized TiO_2_ nanobelts	hydrothermal process	nanobelts diameter: 50–200 nm; length: several micrometers; nanospheres diameter: ~400 nm	ethanol	100–800 ppm	320	R_g_/R_a_: 27.5		400 ppm	[105]
Ag-TiO_2_/SnO_2_ nanocomposites	chemical deposition	diameter: ~100 nm	ethanol	1–500 ppm	275	R_a_/R_g_: 53	3.5/7 s	50 ppm	[104]
TiO_2_/V_2_O_5_ nanoheterostructures	electrospinning	nanobranches diameter: 15–20 nm; nanofibers diameter: 160 nm	ethanol	20–1000 ppm	350	R_a_/R_g_: 24.6	6/7 s	100 ppm	[97]
TiO_2_/Ag_0.35_V_2_O_5_ branched nanoheterostructures	electrospinning	nanobranches diameter: ~20 nm; nanofibers diameter: ~190 nm	ethanol	20–1000 ppm	350	R_a_/R_g_: 31.8	7/12 s	100 ppm	[95]
brush-like ZnO-TiO_2_ heterojunctions nanofibers	electrospinning + hydrothermal process	ZnO nanorods diameter: 100–300 nm; TiO_2_ nanofibers diameter: 100 nm	ethanol	20–500 ppm	320	R_a_/R_g_: 50.6	5/10 s	500 ppm	[110]
TiO_2_/ZnO core-shell nanorods	hydrothermal method + ALD	core width: 120 nm; shell width: 20 nm	ethanol	5–25 ppm	150	R_a_/R_g_: 2.37	100/70 s	10 ppm	[64]
ZnO surface functionalized TiO_2_	electrospinning + hydrothermal treatment	nanofibers diameter : 70–100 nm; ZnO nanosheets diameter: 500 nm	ethanol	10–200 ppm	280	R_a_/R_g_: 15.7	5/3 s	100 ppm	[111]
ZnO/TiO_2_ nanocomposites	CVD		ethanol		400	R_a_/R_g_: 5	~1/1 min	50 ppm	[46]
brookite TiO_2_ decorated a-Fe_2_O_3_ nanoheterostructures	chemical deposition	length: 50–100 nm; diameter: ~10 nm	butanol	10–500 ppm	370	R_a_/R_g_: 27.6	5/6 s	100 ppm	[65]
ZnO-TiO_2_ nanocomposites	CVD	diameter: 5–20 nm	acetone	20–100 ppm	350	R_a_/R_g_: 22.7	1/1 min	100 ppm	[46]
ZnO/TiO_2_ nanocomposites	CVD		acetone			R_a_/R_g_: 22		100 ppm	[46]
CuO-TiO_2_ heterostructure nanofibers	electrospinning + hydrothermal process	TiO_2_ nanofibers: length: several tens micrometers; diameter: 200 nm; CuO nanocubes: diameter: 40–80 nm	formaldehyde	5–100 ppm	200	R_a_/R_g_: 15.5		50 ppm	[112]
Cd/SnO_2_/TiO_2_ composites	sol-gel		formaldehyde	100–500 ppm	320	R_a_/R_g_: 32	25/17 s	200 ppm	[113]
*α*-Fe_2_O_3_/TiO_2_ branch-like hierarchical heterostructures	electrospinning + hydrothermal method	diameter: 600 nm; length: several micrometers	trimethylamine	10–200 ppm	250	R_a_/R_g_: 13.9	0.5/1.5 s	50 ppm	[62]
hierarchically assembled ZnO nanorods on TiO_2_ nanobelts	hydrothermal process	TiO_2_ nanobelts: width: 50–200 nm; length: several micrometers; ZnO nanorods: length: 500 nm	trimethylamine	5–500 ppm	200	R_a_/R_g_: 25		5 ppm	[114]
SnO_2_/TiO_2_ composites	sol-gel		methanol	50–400 ppm	360	R_a_/R_g_: 60	10–15/14–20 s	200 ppm	[115]
ZnO-TiO_2_ nanocomposites	physical mixture		humidity	5%–90% RH	RT (room temperature)	(ΔR)/(Δ%RH): 9.08 MΩ/%RH			[59]
LiCl/TiO_2_ electrospun nanofibers	electrospinning	diameter: 150–260 nm	humidity	11%–95% RH	RT	R_a_/R_g_: 10^3^	<3/7 s	11%–95% RH	[100]
ZnSnO_3_ nanoneedles/TiO_2_ nanofibers heterojunction	electrospinning + hydrothermal treatment	TiO_2_ nanofibers diameters: 200–300 nm; ZnSnO_3_ nanorods: tip diameter: 0.5~1.5 nm; ratio of length to diameter: ~9.6	humidity	11%–95%	RT		2.5/3 s		[116]
ZnSnO_3_ nanoparticles/TiO_2_ nanofibers heterojunction	electrospinning + hydrothermal treatment	TiO_2_ nanofibers diameters: 200–300 nm; ZnSnO_3_ nanoparticles diameters: 30–50 nm	humidity	11%–95%	RT		3.5/29 s		[116]
Ce_2_O_3_/TiO_2_/SnO_2_ thin film	sol-gel		humidity	15%–95% RH	RT	R_a_/R_g_: 100		40%	[117]
polypyrrole-coated TiO_2_/ZnO nanofibers	electrospinning + chemical deposition	TiO_2_/ZnO core diameter: 100 nm; PPy shell thickness: 7 nm	NH_3_	0.5–450	RT	ΔR/R_a_: 0.35		450 ppm	[118]
nanocrystalline TiO_2_/SnO_2_ composites	commercial powder		NH_3_	100–5000 ppm	400	ΔR/R_a_: 0.5		1200 ppm	[119]
SnO_2_/TiO_2_nanoneedles	wet chemical method	diameter: 40–80 nm; length: 60–100 nm	NH_3_		150	(ΔR/R_g_) × 100%: 300%	3/5 min	1000 ppm	[120]
TiO_2_/SnO_2_ thick film	sol		NH_3_	100–1000 ppm	250	R_a_/R_g_: 3		400 ppm	[121]
TiO_2_/ZnO inner/outer double-layer hollow fibers	electrospinning + ALD	inner diameter: ~320 nm; TiO_2_ layer thickness: ~30 nm; ZnO outer layer thickness: 20 nm	CO	0.1–10 ppm	375	R_a_/R_g_: 20.3		1 ppm	[72]
TiO_2_/Fe_2_O_3_ nanosized thin film	sputtering	diameter: 20–30 nm	CO			ΔR/R_a_: 15	~50/- s	1000 ppm	[122]
TiO_2_/Al_2_O_3_/Pd composites	sol		H_2_S	200–1000 ppm	225	log(R_a_/R_g_): 0.9		1000 ppm	[48]
nanocrystalline CdO/ZnO/TiO_2_	pyrolyzation		H_2_S		225–250	ΔR/R_a_: 0.8		10,000 ppm	[123]
TiO_2_ decorated CuO nanorods	thermal evaporative + sputtering	diameters: 50–100 nm; lengths: a few tens of micrometers	H_2_	0.1–5 ppm	300	R_a_/R_g_: 8.57		5 ppm	[124]
TiO_2_/SnO_2_ nanocomposites	physical mixture	diameter: 8–28 nm	H_2_	50–3000 ppm	375				[76]
TiO_2_ fibers supported *β*-FeOOH nanostructures	electrospinning + hydrothermal method	nanofiber diameter: ~500 nm	H_2_	100 500 ppm	RT	R_a_/R_g_: 52.5		500 ppm	[87]
mesoporous Nb_2_O_5_/TiO_2_	sol-gel	diameter: 4.1 nm	H_2_		450	R_a_/R_g_: ~5.5	~1/1 min	500 ppm	[125]
TiO_2_/NiO thin film	sputtering		H_2_	200 ppm–0.5%	300	R_g_/R_a_: 15	2/2.3 min	1000 ppm	[126]
PtO/Pt/TiO_2_ thin film	sol-gel		H_2_	1%–10%	180	ΔR/R_a_ × 100%: 40%	~10/10 min	2%	[103]
TiO_2_-In_2_O_3_ composite nanofibers	electrospinning	diameters: 250 nm; length: several micrometers	NO_2_	0.3–97 ppm	RT	ΔR/R_a_: ~1.25	~ 9.5/- s	1 ppm	[61]
SnO_2_-core/V_2_O_5_-shell nanorods	thermal evaporation + sputtering	length: several tens micrometers; SnO_2_-core thicknesses: 100 nm; V_2_O_5_-shell thickness: 10 nm	NO_2_	10–80 ppm	300	R_g_/R_a_: 1.03%	≤4.5/4.5 min	10 ppm	[127]
Al_2_O_3_ decorated anatase TiO_2_ nanotubes	electrochemical anodization + thermal decomposition	nanotube outer diameter: ≤200 nm; length: several micrometers	NO_x_	0.97–97 ppm	RT	ΔR/R_a_: 88.04%	8/- s	97 ppm	[45]
CuO-TiO_2_-Au nanosystems	CVD + sputtering		O_3_					300 ppb	[71]
V_2_O_5_/TiO_2_ thin film	sol-gel	3–5 nm	O_2_	1 ppm–20.9%	250	R_g_/R_a_: 3.5	5/30 min	120 ppm	[128]
CeO_2_/TiO_2_ thin film	sol-gel		O_2_	5–10,000 ppm	420	R_g_/R_a_: ~3	40–60/80	1000 ppm	[129]
SnO_2_/TiO_2_ thin film	sputtering	44–67 nm	O_2_	100–2000 ppm		R_g_/R_a_: 2		1000 ppm	[130]

**Table 2:** Summary of the gas sensing properties of carbon-group-materials/TiO_2_ nanoheterostructure sensors.

TiO_2_-Based Nanoheterostructures	Fabrication Method	Size	Detection Gas	Detection Range	Response				Ref.
					Operation Temperature (°C)	Sensitivity	Response/Recovery Time	Concentration	
Pd/TiO_2_/reduced graphene oxide ternary composite	one-pot polyol		NH_3_	5–150 ppm	RT	(ΔR/R_a_) × 100%: 39.9%		100 ppm	[142]
PPy/graphene nanoplatelets decorated TiO_2_ nanoparticles	sol-gel + chemical polymerization	TiO_2_ nanoparticles diameter: 10–30 nm	NH_3_	1–200 ppm	RT	(ΔR/R_a_) × 100%: 102.2%	36/16 s	50 ppm	[146]
CNTs/TiO_2_ nanocomposites	screen-printing + dip-coating techniques		NH_3_		RT	ΔR/R_a_: 93	9/2 min	1%	[148]
Pt/TiO_2_/MWCNTs nanocomposites	sol-gel		H_2_	5%–100%	50	(ΔR/R_a_) × 100%: 30%		70%	[149]
CNTs/Pt-TiO_2_ NTs	anodization	diameter: 100 nm;length: 14 um	H_2_	0.5%–3%	100	(ΔR/R_a_) × 100%: 2%		1%	[150]
Pt-TiO_2_/MWCNTs hybrid composites	wet chemical procedure		H_2_	0.5%–3%	150			0.5%	[140]
rGO/TiO_2_ thin film			formaldehyde	0.1–1 ppm	RT	(ΔR/R_a_) × 100%: 0.64	70/126 s	1 ppm	[75]
MWCNTs/TiO_2_ nanocomposites	sol-gel	diameter: 20–40 nm	CO		350	R_a_/R_g_: 15.8	4/16 s	50 ppm	[151]
MWCNTs/TiO_2_ thin film	sol-gel		CO		400	R_a_/R_g_: 89.2	5.16/2.72 s	100 ppm	[152]
graphene-TiO_2_ nanocomposite	sol–gel	TiO_2_ nanoparticles: ~35 nm	CO_2_	500–15,000 ppm	200	R_g_/R_a_: 1.34		10,000 ppm	[144]
TiO_2_/carbon black	sol-gel		NO_2_	1–100 ppm	150	ΔR/R_a_ × 100%: 7%		100 ppm	[153]
single-walled carbon nanotube/TiO_2_ hybrid		length of carbon nanotubes: 20–50 nm	NO	50 ppb–1 ppm	RT	(ΔR/R_a_) × 100%: 9%		50 ppb	[140]
CNT/TiO_2_ hybrid films	sol-gel		O_2_	10 ppm	350	ΔR/R_a_: 6.5	8/- s	10 ppm in CO_2_	[154]
grapheme oxide/nano-anatase TiO_2_		~5 nm	humidity	35%–95%		power loss/ΔRH: ~0.47 dB/%RH	0.74/0.91 s		[141]

**Table 3:** Summary of the gas sensing properties of TiO_2_-based organic/inorganic nanoheterostructure sensors.

TiO_2_-Based Nanoheterostructures	Fabrication Method	Size	Detection Gas	Detection Range	Response				Ref.
					Operation Temperature (°C)	Sensitivity	Response/Recovery Time	Concentration	
TiO_2_/PPy nanocomposites	in situ chemical polymerization	33–67 nm	NH_3_	20–140 ppm	RT	(ΔR/R_a_) × 100%: 7.95%	19/85 s	141 ppm	[88]
PPy-coated TiO_2_/ZnO nanofibers	electrospinning + chemical deposition	TiO_2_/ZnO core diameter: 100 nm; PPy shell thickness: 7 nm	NH_3_	0.5–450 ppm	RT	ΔR/R_a_: 0.35		450 ppm	[118]
PPy/TiO_2_ nanocomposites	layer by layer self-assembly technology.		NH_3_	10–1600 ppm	RT	frequency shift (ΔF): 50 Hz	~100/200 s	10 ppm	[165]
PPy/TiO_2_	in situ polymerization		NH_3_	20–500 ppm	RT	ΔR/R_a_: 0.13		100 ppm	[169]
PANi/TiO_2_ nanofibers	electrospinning	diameter: 600 nm	NH_3_	>50 ppt	RT	ΔR/Ra: 0.018	<10/10 s	200 ppt	[49]
PANi/TiO_2_ thin film heterojunction	chemical polymerization + sol-gel		NH_3_	20–100 ppm	RT	(ΔR/R_a_) × 100%: ~11%	41/- s	100 ppm	[74]
polyaniline/TiO_2_ nanorods heterostructure	hydrothermal method		NH_3_	5–100 ppm	RT	(R_g_/R_a_) × 100%: 610%	40/60 s	100 ppm	[167]
cellulose/TiO_2_/PANi composite nanofibers	electrospinning		NH_3_	10–250 ppm	RT	ΔR/R_a_: 0.584		10 ppm	[168]
TiO_2_-PANi/PA6 nanofibers	electrospinning + sputtering		NH_3_	50–250 ppm	RT	ΔR/R_a_: 18.3	<50/50 s	250 ppm	[170]
PANi/TiO_2_ nanocomposite thin film	in situ self-assembly technique	diameter: 90 nm	NH_3_	20–140 ppm	RT	ΔR/R_a_: 0.3	2–3/~60 s	1 ppm	[88]
CSA/PANi/TiO_2_ thin film	sol-gel		NH_3_	20–100 ppm	RT	ΔR/R_a_: 0.75	49/413 s	100 ppm	[171]
TiO_2_–PANi nanocomposite thin film	spin coating	~20 nm	CO_2_	53–1000 ppm	RT	R_g_/R_a_: 53	9.2/5.7 min	1000 ppm	[164]
PANi doped TiO_2_ nanocomposite thin film	spin coating	21 nm	LPG		RT	R_g_/R_a_: 2.37	2.6/2.4 min	2000 ppm	[164]
TiO_2_/PPy/poly 3-[(methacryoylamino) propyl trimethylammonium chloride] (PMAPTAC) nanocomposite thin film	in situ photopolymerization		humidity	13–90% RH	RT	log Z: ~6	30/45 s	60%	[172]
TiO_2_/PPy nanocomposite film	in situ photopolymerization		humidity	30–84% RH	RT	log Z: ~5.5	40/20 s	30%	[173]
